# Correlates of age at first birth among women in Ethiopia: use of multilevel survival analysis models

**DOI:** 10.11604/pamj.2023.44.190.36090

**Published:** 2023-04-20

**Authors:** Essey Kebede Muluneh, Mahider Alemu

**Affiliations:** 1 School of Public Health, Bahir Dar University, Bahir Dar, Ethiopia,; 2 Department of Statistics, Woldia University, Woldia, Ethiopia

**Keywords:** Women, multilevel, survival analysis, frailty models, Ethiopia

## Abstract

**Introduction:**

the timing of birth of the first child has a direct relationship with fertility in general and health and future career including further education of a mother in particular. The objective of this study was to identify factors significantly associated with the time to the first birth among women in Ethiopia.

**Methods:**

a cross-sectional study was conducted using data from the 2016 Ethiopian Demographic and Health Survey (EDHS). The study subjects were married women and men aged 15 to 49 in randomly selected households across Ethiopia and two stage stratified random sampling technique was used to select study subjects. Log logistic-Gamma shared frailty model was used to identify factors associated with the length of time spent until the first birth.

**Results:**

the median age at first birth for women living in Ethiopia was 20 years, whereas the minimum and maximum ages at first birth were 11 and 49 years respectively. Age at first sex, age at first cohabitation, sex of household head, place of residence, religion, education level, contraceptive use and exposure to media were significant correlates of age at first birth of women in Ethiopia. Higher level of education was associated with increased age at first birth. Women who use contraceptive, women living in urban areas, women having exposure to media and female headed households had longer time to first birth compared to their counterparts.

**Conclusion:**

the different regions of Ethiopia have significant differences in the age of women during their first birth. Most of the factors associated with the time to first child in this study were related to education of women. Investing in education and educating women plays critical roles in regulating fertility of a nation and health of women.

## Introduction

The first evident outcome of the fertility process is the birth of the first child. The first birth marks a woman´s transition into motherhood and signifies her on her future life and her roles and responsibilities. In the absence of any active fertility control, the age at which childbearing begins influences the total number of children a woman bears throughout her reproductive period, the cumulative effect being rapid population growth. There exists strong relationship between age at first birth and total fertility: the earlier a woman experiences her first birth, the more children she will have. For countries in sub-Saharan Africa, where contraceptive use is relatively low as compared with the developed world, younger age at first birth has strong effects on both individual and aggregate levels of fertility. Total fertility declines with increasing age at first birth [1-3]. Age of a woman at her first birth is related not only to the number of children she will have but also to the health and well-being of the mother. An early age at first birth often has a negative effect on further education and career-building, marital stability, asset accumulation, and on the woman's health. Girls who gave birth before the age of 18 were disproportionately affected by complicated maternal mortality and morbidity. Girls aged 10 - 14 years and girls aged 15 - 19 years were five times and twice respectively more likely to die in pregnancy or childbirth than women aged 20 – 24 [4-9].

Several factors such as parents´ socioeconomic status, education, wealth index, marital status, biological factors, region of birth, rural/urban origins and parents´ religion are assumed to affect the age of the woman giving birth for the first time were identified as significant determinants of the timing of the first birth [2,10]. Level of education is correlated with age at first birth - the least educated women entering earlier into motherhood. Education and early fertility are known to be negatively correlated. On the one hand, early fertility often leads to termination of schooling. On the other hand, education can affect women´s early fertility decisions through access to knowledge, information, and changes her attitudes and behavior [11-13].

It is expected that women who grew up in a rural setting will become mothers at a younger age than those who did not. Early motherhood may seem more attractive than pursuing a career when educational opportunities are limited, as they are in many rural areas [7,14] Ethiopian social norms and family policies can influence the timing of a first birth [15]. A study in Uganda [16] reported that age of respondent, education, religion, region, place of residence (Rural/Urban), and age at first sexual intercourse were the most important determinants of age at first birth.

Even though the issue of mothers´ age at their first birth has wide-ranging consequences on total fertility and health of mothers, not enough research has been conducted to identify its determinant factors. In an attempt to identify factors influencing the timing of the first birth, use of appropriate statistical analysis method plays a considerable role. Several statistical methods have been used in studies aimed at identifying correlates of age at first birth [1,2,13,16-19]. Since the response variable of interest is waiting time to the occurrence of an event (birth in this case) survival analysis technique is the preferred method unless the time is recorded in age groups, in which case the use of logistic regression may be an option [13,20]. Most studies have employed standard survival analysis methods like the Cox-proportional hazards (PH) model [18,19,21]. However, the model has constantly been criticized for its restrictive assumption commonly referred to as the proportional hazards (PH) assumption [21,22].

Data with a multilevel or hierarchical structure occur frequently in a wide range of research disciplines, including epidemiology, health services research, public health, education and sociology. When we have clustered data or subjects nested within some other hierarchy or repeated measures on a subject, individuals belonging to the same group are usually correlated and hence the assumption of independent observations in the modeling process does not hold. Multilevel survival models are used to model such types of survival data [22]. In view of the socioeconomic, cultural and environmental differences among the different regions (states) in Ethiopia, the objective of this study was to assess the presence of regional variation in age at first birth and identify factors significantly associated with time-to-first birth for Ethiopian women using multilevel survival models. These models help to account for the loss of independence that arises from the clustering of women in the different regions (states) of Ethiopia.

## Methods

**The data:** the dataset used in this study was obtained from the 2016 Ethiopian Demographic and Health Survey (EDHS). The survey was conducted by the Central Statistics Agency (CSA) from January 18, 2016, to June 27, 2016, based on a nationally representative sample that provides estimates at the national and regional levels and for urban and rural areas. The survey target groups were women aged 15-59 in randomly selected households across Ethiopia.

**Study design:** this study used a quantitative, cross-sectional design by analyzing data from the 2016 Ethiopia Demographic and Health Survey (EDHS).

### Sample design

Ethiopia is constitutionally formed as a federation of nine ethnically-based regional states (regions) and two chartered cities. These eleven administrative regions have basic roles of managing public health services and coordinating the operation of health system under their jurisdiction. The 2016 EDHS sample was selected using two-stage cluster design. Census enumeration areas (EAs) were the sampling units for the first stage. The sample included 645 EAs (202 in urban and 443 in rural areas). Households comprised the second stage of sampling. A complete listing of households was carried out in each of the selected EAs. A representative sample of 17,067 households was selected for the 2016 EDHS. Complete interviews were conducted for 15,683 eligible women of ages 15-49 years. This group of women having complete data comprised the sample for this study. To get unbiased estimates for population parameters in complex study settings like the 2016 EDHS sample which used a two-stage stratified cluster sampling technique, sampling weights based on sampling probabilities separately for each sampling stage and each cluster were used.

### Data analysis

The response variable in this study was the age of the mother at her first birth. It is measured as the length of time from her date of birth until the age at which she gives her first birth which is measured in years and is assumed to be continuous. If a woman did not give birth until the date of the data collection, the data is said to be right censored. If, however, a woman has given birth until the date of the data collection, we say the event (first birth) has occurred. This kind of data, where the duration of time until some kind of event occurs is of interest, is called survival data or time-to-event data. The independent variables that were known to be associated with the timing of first birth and considered in this study are sex of household head, wealth index, religion, education level, place of residence, contraceptive use, respondents´ working status, media exposure, alcohol consumption, number of siblings, age at first sexual encounter and age at first cohabitation. Survival data analysis is the appropriate method of dealing with time-to-event data. Parametric and non-parametric approaches of analyzing survival data were applied in this study. Descriptive statistics were produced for the different categories of women. The non-parametric methods (Kaplan Meier curves and the log rank test) were used to compare the ages at first birth of women in different categories of the factors. The Cox proportional hazards (PH) regression model is a broadly applicable and most widely used method of survival analysis. Important assumptions used in this model are that the observations from different subjects are statistically independent of each other and that the relative hazard (hazards ratio) does not change over time. This is known as the proportional hazards assumption.

Researchers often encounter grouped or multilevel data like individuals nested within families, and families nested within neighborhoods. For instance, women aged 15-49 are nested within regions. Most multivariate models assume that observations are independent, while grouped data clearly violate this assumption. For example, as in this study, populations in adjacent areas are likely to display similar behavior, which can influence timing of first birth and causing it to be spatially auto-correlated. While it is commonly assumed that there is independence between the observations from different clusters, it will be unreasonable to assume that observations in a cluster are independent. Failure to take into account this clustering from such hierarchical populations typically leads to underestimation of standard errors. Multilevel survival analysis using mixed effects Cox regression model is used to model survival data when repeated measures are taken for each individual or the survival times are clustered (grouped) or individuals are nested within some other hierarchy, or some other reason to have both fixed and random effects [23]. Parametric shared frailty model which assumes a parametric distribution for baseline hazard function is a special case of mixed effects Cox regression models [24-26]. Mixed effects Cox regression model is formulated as


hit=hotexpxi'β+αj

where α_j_ denotes the random effects associated with the j^th^ cluster, h_o_ (t) is the baseline hazard function, and X_i_' = (X_i1_, X_i2_, X_i3_...,X_ip_) is the vector of independent variables (factors). The term ‘shared frailty’ is used to denote the exponential of the random effect exp(α_j_). The random effect can be thought of as a random intercept that modifies the linear predictor, while the shared frailty term has a multiplicative effect on the baseline hazard function [25]:


hit=h0texpαjexpxi'β

Expanding proportional hazards model to include a random effect, called a frailty, allows for modeling the association between individual failure times within a group. A frailty acts multiplicatively on the hazard function and the model that incorporates this random effect into the hazard function is called the frailty model [25]. Suppose there are G groups with *n_i_* individuals in the *i^th^* group; X_ij_ is the observable covariate vector for the j^th^ individual in the *i^th^* group. Let ω_i_ be the unobservable covariates for the *i^th^* group and *ψ* be the regression coefficient of the unknown covariates. The hazard function of the j^th^ individual in the *i^th^* group is [27].


$$h_{ij}\left(t|X_{ij}\right)=h_{0}\left(t\right)\exp\left(\beta X_{ij}+\Psi w_{i}\right),i=1,\ldots \ldots ,n_{i}$$

Akaike´s information criterion (AIC) was used for model comparison. Data analysis were carried out using STATA, Version 12. All hypotheses testing to determine differences, associations and relationships were judged significant at p < 0.05.

**Ethical considerations:** the ethical clearance for the survey was approved by the Ethical Review Board of Central Statistical Agency (CSA) of Ethiopia and all participants who agreed to take part in the survey and signed an informed consent form to participate in the study were included.

## Results

### Descriptive statistics

Of all the 15683 women aged 15-49, 10274 (65.5%) had at least one child until the date of data collection. The median age at first birth was 20 years, while the minimum and maximum ages at first birth were 11 years and 49 years respectively. The median age at first birth is highest in the capital city Addis Ababa (27 years) and lowest in Amhara, Afar, Oromia, Benishangul, and Gambella regions (19 years each). The median ages at first birth for rural and urban women were respectively 19 years and 23 years. Median age at first birth increases continuously as the level of education of mothers increases; 28 years for women who have attained higher education and 19 years for women with no formal education. Muslim women tend to have lower median age at first birth (19 years) as compared with women following Orthodox Christianity in Ethiopia (21 years). The percentage of rural women who had at least one child until the end of the study period (73%) was higher than their counterparts in the urban (51%). Afar region has the highest percentage of women who had at least one child until the end of the study period (74.02) while Addis Ababa city had the least (41.67). Eighty-eight percent of women with no formal education had at least one child during the study period. The corresponding figures for women having secondary and higher education were 39% and 40% respectively (Table 1).

**Table 1:** demographic characteristics of women at first birth, EDHS 2016

Variable	Category	Total number of Women	Percent of women who gave Birth	Median age at first birth (years)
Region	Tigray	1682	1107(65.81)	20
	Afar	1128	835(74.02)	19
	Amhara	1719	1160(67.48)	19
	Oromia	1892	1366(72.20)	19
	Somali	1391	1002(72.03)	20
	Benishangul	1126	804(71.40)	19
	SNNPR	1849	1225(66.25)	20
	Gambella	1035	756(73.04)	19
	Harari	906	605(66.78)	20
	Addis Ababa	1824	760(41.67)	27
	Dire Dawa	1131	654(57.82)	22
Sex of Household Head	Male	10853	7438(68.53)	20
	Female	4830	2836(58.72)	21
Wealth Index	Poorest	3894	3029(77.79)	19
	Poorer	2046	1548(75.66)	19
	Middle	2002	1400(69.93)	19
	Richer	2042	1361(66.65)	19
	Richest	5699	2936(51.52)	23
Religion	Orthodox	6413	3829(59.71)	21
	Catholic	91	58(63.74)	20
	Protestant	2814	1871(66.49)	20
	Muslim	6209	4383(70.59)	19
	Other	156	133(85.26)	19
Highest Education Level	No Education	7033	6190(88.01)	19
	Primary	5213	2731(52.39)	20
	Secondary	2238	873(39.01)	25
	Higher	1199	480(40.03)	28
Type of Place of Residence	Urban	5348	2732(51.08)	23
	Rural	10335	7542(72.98)	19
Contraceptive Use	Yes	3312	2920(88.16)	19
	No	12371	7354(59.45)	20
Respondents Working Status	No	10011	6589(65.82)	20
	Yes	5672	3685(64.97)	21
Media Exposure	No	8127	6200(76.29)	19
	Yes	7556	4074(53.92)	22
Drinking Alcohol	No	10516	6954(66.13)	20
	Yes	5167	3320(64.25)	20

### Kaplan-Meier (KM) survival curves for different groups

**Figure 1:**
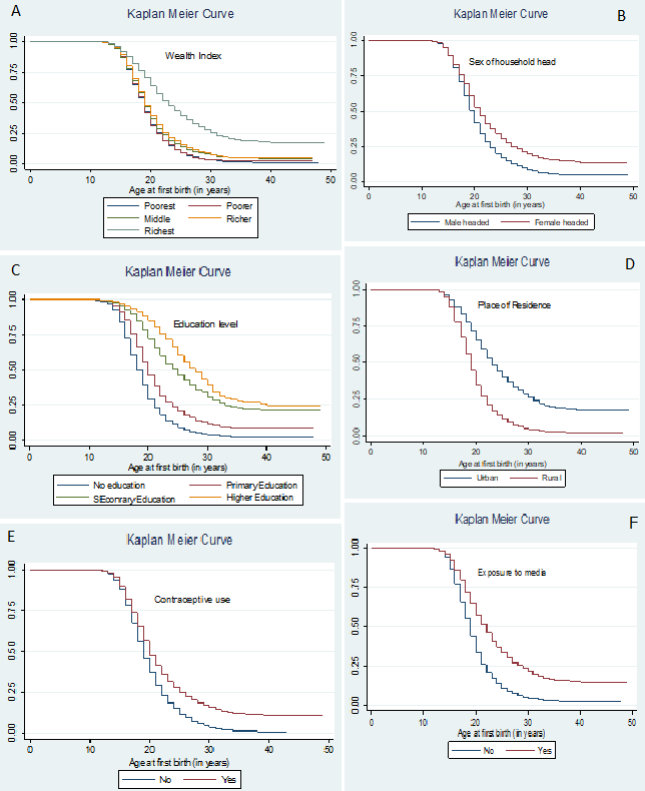
(A-F) survival probability curves of time to first birth (in years) versus different categories of socioeconomic factors

The Kaplan-Meier estimate of the survival curve is the best description of times to event of a group of subjects as it uses all the data currently available. The resulting KM survival curve based on EDHS 2016 dataset is shown in the following figures. Note that in this plot of survival time is being measured in years (Figure 1 (A-E)). From the Kaplan Meier probability curves in Figure 1 (A-F), we can observe that the survival curves of richest women, women residing in urban areas, women who have attained higher education, women in the female-headed households, women having exposure to media and contraceptive user women lie above the curves of other groups of mothers implying that these groups of mothers had relatively older age at first birth. The results of the log-rank test displayed in Table 2 also show that age at first birth of Ethiopian women shows variability among the different categories of these and other factors (Table 2).

**Table 2:** results of the log-rank test for each categorical variables of age at first birth, EDHS 2016

Covariate	Chi-square	DF	P-value
Region	1202.84	10	0.0000
Type of place of residence	1456.36	1	0.0000
Wealth index of house hold	1482.48	4	0.0000
Sex of household head	248.29	1	0.0000
Religion	343.67	4	0.0000
Education level	2366.08	3	0.0000
Drinking alcohol	87.28	1	0.0000
Media exposure	1107.27	1	0.0000
Respondent working status	173.57	1	0.0000

### Statistical analysis

Based on the nature of the data, several models were fitted and compared. A univariable analysis was performed for each potential model to see the effect of each explanatory variable on age at first birth and to select variables to be included in the multivariable analysis. To adjust for confounding, all factors which were independently associated with the time to the first birth in the univariable analysis were included in the multivariable analysis and comparison was done among the models using Akaike Information Criteria (AIC) value, where the model with minimum AIC value is considered a better fit for the data [28] (Table 3). In all the multivariable parametric gamma shared frailty models, educational level of women was significant, indicating that it was the most important determinant factor for the time to age at first birth based on EDHS 2016 dataset. Age at first sexual encounter, age at first cohabitation, religion, place of residence (rural/urban) and use of any contraceptive method were also significant factors for all parametric gamma shared frailty models. Sex of household head and alcohol consumption were significant correlates of age at first birth for log logistic-gamma and Weibull-gamma shared frailty models. The number of siblings and wealth index were significant factors for Weibull-gamma shared frailty model. Access to media was significant factor for log logistic gamma shared frailty model. Working status of women was not a significant determinant of time to age at first birth using all models based on EDHS 2016 dataset.

**Table 3:** AIC value and test of unobserved heterogeneity for multivariable parametric gamma shared frailty models with different baseline distributions, EDHS 2016

Gamma Shared frailty model	AIC	P-value
Exponential-Gamma	26775.554	<0.001
**Log logistic-Gamma**	**2456.1928**	**<0.001**
Weibull-Gamma	7239.219	<0.001
Log logistic AFT model	3619.9808	<0.001
Log logistic gamma shared frailty model	2456.1928	<0.001
Complementary log-log binomial linear model with mixed effects model	10,699.5026	<0.001

The variance of the random effect (θ) was significant for all multivariable parametric gamma shared frailty models implying that there is significant variation in the ages at first birth of women from different regions. It was highest when we assume the log logistic-gamma shared frailty model (θ=0.585). The Kendall´s tau (τ) was highest for the highest θ values. Accordingly, the dependency within the clusters for log logistic-gamma shared frailty model (τ=0.226) was the maximum. Based on the AIC criteria (the model with the smallest value is the best model), the log-logistic gamma shared frailty model, having the AIC value of 2456.1928, was the preferred model for the dataset under consideration. Results of log logistic gamma shared frailty model showed that age at first sex, age at first cohabitation, sex of household head, place of residence, religion, education level, contraceptive use, alcohol consumption, and media exposure were the important correlates (factors) for the time to age at first birth (Table 4). The value of the shape parameter in the Log logistic-Gamma shared frailty model was (γ=0.086). This indicates non-monotonic hazard rates, specifically initially increasing and then decreasing rates. That means the risk of giving birth tends to increase when the age increases until some time and finally decreases when the mother gets older. The estimated value of theta (θ) is 0.585.

A variance of zero (?=0) would indicate that the frailty component does not contribute to the model, meaning that age at first birth does not vary among the different regions in Ethiopia. The likelihood ratio test for the hypothesis ?=0 presented at the bottom of Table 4 shows a chi-square value of 1140.90 with one degree of freedom (p-value < 0.0001) which is highly significant indicating that the frailty component had significant contribution to the model. This shows that age at first birth varies among regions in Ethiopia. The dependence within region was τ=22.6%.

**Table 4:** multivariable analysis using the Log logistic-Gamma shared frailty model, EDHS 2016

Covariate	Coeff	St.err	P-value	ϕ	[95% CI for ϕ]
Constant	2.8927	0.0103	0.000	18.0419	17.6815 18.4098
Age at first sex	-0.0027	0.0007	0.000	0.9973	0.9959 0.9987
Age at first cohabitation	-0.0014	0.0007	0.036	0.9986	0.9973 0.9998
Sex of household head					
Male (ref)					
Female	0.0096	0.0044	0.027	1.0096	1.0011 1.0183
Place of residence					
Urban (ref)					
Rural	-0.0521	0.0054	0.000	0.9492	0.9392 0.9594
Religion					
Orthodox (ref)					
Catholic	0.0059	0.0244	0.807	1.0059	0.9590 1.0552
Protestant	-0.0032	0.0072	0.661	0.9968	0.9828 1.0110
Muslim	-0.0428	0.0066	0.000	0.9581	0.9458 0.9706
Other	-0.0066	0.0173	0.703	0.9934	0.9602 1.0278
Highest education level					
No education (ref)					
Primary	0.0615	0.0039	0.000	1.0635	1.0552 1.0718
Secondary	0.1698	0.0062	0.000	1.1850	1.1706 1.1996
Higher	0.2739	0.0083	0.000	1.3150	1.2939 1.3366
Contraceptive use					
No (ref)	0.0489	0.0047	0.000	1.0501	1.0406 1.0598
Yes					
Media exposure No (ref) Yes	0.0387	0.0043	0.000	1.0395	1.0307 1.0484
Drinking alcohol					
No (ref)	-0.0179	0.0061	0.004	0.9823	0.9706 0.9942
Yes					

Φ= 0.585; γ = 0.086; τ= 0.226; Likelihood-ratio test of theta=0: chibar2(01)=1140.90 prob >= chibar2(01) = 0.000;

Analysis based on log logistic-gamma shared frailty model showed that age at first sexual encounter, age at first cohabitation, sex of household head, type of place of residence (rural/urban), religion, highest education level, contraceptive use, alcohol consumption, and exposure to media were significant correlates of age at first birth at 5% level of significance. In contrast respondent´s working status, wealth index and numbers of siblings were not significant at 5% level of significance. Women, who have attended primary school, secondary school and higher education, had significantly different ages at first birth as compared to women with no education. An acceleration factor of greater than 1 indicates prolonging the time to age at first birth. Based on this criterion, women who attended primary school (Φ=1.0635), secondary school (Φ=1.1850), and higher education (Φ=1.3150) had prolonged age at first birth compared with women with no education. Women residing in rural areas of Ethiopia have entered into motherhood earlier than those residing in urban areas of the country (Φ=0.9492). Similarly, adolescents in female headed households have given birth earlier than those male headed households in Ethiopia (Φ=0.9986).

Women using any contraceptive method had significantly different age at first birth than women who were not using contraceptives. The resulting acceleration factor showed that women who had used any contraceptive method had prolonged time at first birth by a factor of 1.0501 than women who have not used any contraceptive method. Regarding media exposure, women who had access to media had delayed time to a first birth than women who had no access to media (accelerating factor = 1.0395). Women who follow Catholicism had prolonged age at first birth as compared to women following Orthodox Christianity while Muslim women enter into motherhood earlier than women in the Orthodox group.

## Discussion

The age at which the first child is born affects the total number of children a woman may have throughout her reproductive period and has many implications for different socioeconomic and demographic outcomes. Early motherhood, in addition to affecting the life of a mother in general, has significant contribution to the aggregate level of fertility and consequent population size at national level. This study applied log logistic-gamma shared frailty model to identify factors associated with the length of time spent until the first birth of Ethiopian women based on the 2016 Ethiopian Demographic and Health Survey (EDHS 2016) data set. A total of 15,683 women of ages 15-49 years having complete interviews were included in the study. The median age at first birth for women living in Ethiopia was 20 years, whereas the minimum and maximum ages at first birth were 11 and 49 years respectively.

We found that place of residence was significant determinant of age at first birth in Ethiopian women; rural women had their first child earlier than urban women. This might be due to the fact that women in rural areas of Ethiopia are less likely to go to school and hence enter in to marriage earlier which results in bearing a child at early age. The institutional and normative structures such as the kinship and extended family and limited maternal health-seeking behavior of women in the rural areas also promote early childbearing. On the other hand, urban women need to pursue their career and gain more resources, and achieve maturity to manage an independent household prior to bearing a child and thus they have prolonged time-to-first birth. They also have a better access to maternal health and family planning programs as compared to rural women. The results are in line with studies [13,16,29,30] which indicated that women who are living in rural areas had a higher risk of early first birth than those in urban areas.

Women´s level of education affects their knowledge and usage of modern contraceptive methods, and delays entry into marriage, which reduces the exposure time to risk of childbearing. This is also linked to women´s working status. The educational level of women had a significant effect on time to first birth and it prolonged age at first birth by a factor of Φ=1.0635, Φ=1.1850 and Φ=1.3150 for primary, secondary and higher education respectively compared to illiterate women meaning that women who have attained higher education are more likely to have delayed entry to motherhood compared with those with lower level of education or no education at all. A study on adolescent first births in three East African countries; Uganda, Kenya and Tanzania also revealed that adolescent first births, particularly at the youngest ages, are most common among the poorest and least educated [13]. A number of other studies have similarly observed a positive relationship between women´s educational achievement and age at first birth [7,17,31-34]. A study in Bangladesh [35] reported however that women who were not educated gave birth earlier than educated women.

We found in this study that Catholic women had prolonged age at first birth compared with followers of the Ethiopian Orthodox church while women who follow Muslim, Protestant and other religions had shorter age at first birth. This finding was consistent with a study in Uganda by Mugarura *et al*. [16]. As expected and consistent with many studies such as those in Nigeria, Cameroon and Portugal [34,36,37], use of contraceptive methods before the birth of the first child brings a considerable reduction in the risk of having the first child early. Contraceptive user women had older age at first birth (a factor of Φ=1.0501) compared with those women who did not use any contraceptive. One potential reason, among others, for urban women to have their first child earlier than women in urban areas is the poor access to contraceptives. Age at first sexual experience was also a significant correlate of early pregnancy and this is consistent with other studies conducted in Nigeria, Cameroon, South Africa and Nicaragua [20,34,36,38,39]. This might have happened due to limited utilization of contraception and having limited knowledge on how to prevent pregnancy that happens before maturity or sometimes due to peer influences. The school curricula in Ethiopia do not have enough of reproductive health education component which implies that it did not help adolescents to reduce unprotected sex and its subsequent consequences.

Watching television (TV) and listening to radio at least once a week were considered to measure exposure to media for both women and their partners in the 2016 EDHS. In this study, we found that exposure to media had a significant effect on age at first birth in that women who have access to different media stayed longer to give first birth than women who had no access to media. This is logical as many family planning and maternal and child health awareness creation programs are communicated through mass media. These results are in line with other studies [20,40].

**Study limitations:** some important variables related to male partners were not available which may have effects on the model parameters. In the complementary log-log binomial linear model with mixed effects a large value of AIC was obtained. One important reason may be due to the fact that the dataset was restructured, and the follow-up time (age at first birth) was discretized into interval for as the model requires. Such discretization process may result in a loss of information and may fall short of explaining the hazard of age at first birth by the effect of covariates.

## Conclusion

This study was aimed at identifying correlates of age at first birth among Ethiopian women using survival analysis methods on the 2016 EDHS. It was found that the median age at first birth for the whole nation was 20 years. Women from Addis Ababa city have the largest age at first birth (27 years). The findings from this study showed that there was a clustering (frailty) effect on time to age at first birth among women living in the different regions of Ethiopia implying that women living in the same region share similar risk factors related to birth. Women residing in urban parts of Ethiopia, women having better education and women who were using any contraceptive method had prolonged age at first birth as compared to their men counterparts. Access to media also helps to prolong age at first birth. As level of education increases, the median age at first birth also increases. It should be noted in general that women´s level of education affects their knowledge and usage of modern contraceptive methods, their working status and their ages of entry into marriage, which influence their exposure time to risk of childbearing. Therefore, educating women plays a critical role in regulating the time to the first birth.

### What is known about this topic

- *Multivariate models assuming independence of observations across all regions were used to model similar data; this assumption leads to underestimation of standard errors;*
- *Age at first birth is low in Ethiopia as compared to the global average*.

### What this study adds

- *Regional states have significant variations in age at first birth;*
- *Since women in the same region resemble more than women in other regions clustering among regions was considered and multilevel survival analysis using mixed effects Cox regression model (shared frailty model) was used to model the given data;*
- *Most of the factors associated with the time to the birth of the first child in this study were related to education of women*.
